# Radiologic and histopathologic effects of favipiravir and hydroxychloroquine on fracture healing in rats

**DOI:** 10.1007/s00210-024-03147-y

**Published:** 2024-05-14

**Authors:** Giray Tekçe, Mehmet Arıcan, Zekeriya Okan Karaduman, Yalcın Turhan, Sönmez Sağlam, Mücahid Osman Yücel, Sinem Kantarcıoğlu Coşkun, Cengiz Tuncer, Veysel Uludağ

**Affiliations:** 1 Department of Orthopedics and Traumatology, Faculty of Medicine, Duzce University, 81000 Duzce, Turkey; 2 Department of Pathology, Faculty of Medicine, Duzce University, Duzce, Turkey; 3 Department of Neurosurgery, Faculty of Medicine, Duzce University, Duzce, Turkey

**Keywords:** Fracture healing, Hydroxychloroquine, Rat, Favipiravir

## Abstract

Fracture healing is a process in which many factors interact. In addition to many treatments, physical and biological therapy methods that affect different steps of this process, there are many biological and chemical agents that cause fracture union delay. Although the number of studies on fracture healing is increasing day by day, the mechanism of fracture healing, which is not fully understood, still attracts the attention of all researchers. In this study, we aimed to investigate the effects of favipiravir and hydroxychloroquine used in the treatment of COVID-19. In this study, 48 male Wistar rats weighing 300 ± 50 g were used. Each group was divided into eight subgroups of six rats each to be sacrificed at the 2nd and 4th weeks and evaluated radiologically and histologically. Favipiravir (group 1), hydroxychloroquine (group 2), favipiravir + hydroxychloroquine (group 3), and random control (group 4) were used. A statistically significant difference was observed between the 15th day histological scoring averages of the groups (*p* < 0.05). Although there was no statistically significant difference between the 15th day radiological score distributions of the groups (*p* > 0.05), we obtained different results in terms of complete bone union distributions and radiological images of the fracture line. Although favipiravir has a negative effect on fracture union in the early period, favipiravir may have a positive effect on fracture union in the late period. We did not find any effect of hydroxychloroquine on fracture union.

## Introduction

Bone tissue provides mechanical support to the body; provides movement; protects and supports vital organs such as the brain, heart, and lungs; indirectly produces blood; and stores some minerals because it contains bone marrow (Charoenngam et al. 2023). Today, one of the main subjects of Orthopedics and Traumatology Clinics is bone fractures. Fracture is defined as the complete or partial deterioration of the integrity of the bone and related soft tissues due to internal or external forces (Einhorn & Gerstenfeld 2015). Fracture healing refers to a process in which many factors interact. There are many physical and biological treatment methods that affect different steps of this process (Schmidt et al. 2022). Fracture healing begins as soon as the fracture occurs and continues until the fracture ends join regular bone tissue (Steppe et al. 2023).

Although the number of studies on fracture healing is increasing daily, the mechanism of fracture healing is not fully understood, and the factors affecting this mechanism are still unclear (Alıç et al. 2016). Products that can increase bone metabolism are used to stimulate and activate bone healing in elderly patients and young patients with impaired bone regeneration (Kaiser et al. 2018). The COVID-19 pandemic, which emerged in the last months of 2019, has affected our country as well as the world. The effects of this infection on mortality and morbidity are evaluated in patients with a positive COVID-19 test and fractures (especially hip fractures requiring surgery) via examinations performed on many patients hospitalized for fracture treatment (Egol et al. 2020; Hadfield & Gray 2020; Upadhyaya et al. 2020). Favipiravir and hydroxychloroquine, which are frequently used worldwide and in our country, are beneficial for the treatment of COVID-19, especially in China, Japan, Russia, and our country (Joshi et al. 2021). Favipiravir is a new drug currently generally used for treating influenza virus. In addition, it has an important effect because it directly inhibits the RNA-dependent RNA polymerase enzyme but does not affect cellular RNA or DNA polymerase (Caroline et al. 2014). It is thought that favipiravir will play an important role in the treatment of COVID-19 since COVID-19 is an RNA-dependent RNA polymerase virus (Hanioka et al. 2021; Joshi et al. 2021).

Hydroxychloroquine, another drug frequently used to treat COVID-19 worldwide and in our country, is an anti-inflammatory and immunomodulatory drug that is used safely for treating many rheumatological diseases, such as rheumatoid arthritis, malaria, and systemic lupus erythematosus. Although it is thought that it exerts its immunomodulatory effects by acting on the MHC class 2 antigen, it is currently thought to affect the Toll-like receptor. In vitro studies have revealed the antiviral property of this drug and increased interest in its therapeutic potential against COVID-19 (Gavriatopoulou et al. 2021; Masimirembwa et al. 1994).

In this study, we aimed to examine the effects of favipiravir and hydroxychloroquine, which can be used in the treatment of COVID-19. Radiological and histopathological examination of fracture union in male rats. There is no study in the literature investigating the effects of these drugs on fracture union together, and our study is the first study in the literature to examine both drugs.

## Materials and methods

### The design of the study

In this study, 48 male Wistar-Albino rats (Duzce University, Düzce Medical Faculty Experimental Animals Application and Research Center) were used. Before the study, necessary permission was obtained from the Düzce University, Düzce Medical Faculty Experimental Animals Local Ethics Committee (2021/07/02). The study was carried out at Düzce University, Düzce Medical Faculty Experimental Animals Application and Research Center Laboratory. Laboratory animal care principles were followed in this study. Our study was reported in accordance with the ARRIVE guidelines and the experimental animal ethics committee.

The mean age of the rats included in the study was 2.5 months (2–3 months), and their average weight was 250 g (200–300 g). Animals were randomly divided into four groups (with computer-generated numbers) and followed in the laboratory environment for 1 week before surgery with 12 rats in each cage. In addition, rats of equal weight were selected to avoid weight and obesity factors. During the study, the rats were given unlimited tap water (ad libitum) and standard rodent chow. The animals were monitored in a cage in a room with a controlled temperature (23–25 °C) and a 12:12-h light/dark cycle. Antibiotic prophylaxis was not applied to any group before, during, or after the intervention. Only one rat from the control cage died in the first 24 h after the operation, and the number of animals was completed as of the 1st day of the study; this group did not receive any treatment. No rats died in any group during the remainder of the study.

Postoperatively, 48 rats were treated with favipiravir (group 1, *n* = 12), hydroxychloroquine (group 2, *n* = 12), favipiravir + hydroxychloroquine (group 3, *n* = 12), or control (group 4, *n* = 12) and separated into groups (Table 1).

**Table 1:** Distribution of the experimental animals to be used in the study according to the groups

Groups	Number of rats	Treatment duration (days)	Treatment
Group 1 (control) *n* = 12	6	15	None
	6	30	None
Group 2 (favipiravir) *n* = 12	6	15	Favipiravir ~ 23 mg/kg in the morning and evening; 2–5. Days: 8.5 mg/kg morning and evening (15 days)
	6	30	Favipiravir ~ 23 mg/kg in the morning and evening; 2–5. Days: 8.5 mg/kg morning and evening (30 days)
Group 3 (hydroxychloroquine) *n* = 12	6	15	Hydroxychloroquine 10 mg/kg morning and evening; 2–5. Days: 3 mg/kg morning and evening (15 days)
	6	30	Hydroxychloroquine 10 mg/kg morning and evening; 2–5. Days: 3 mg/kg morning and evening (30 days)
Group 4 (favipiravir + hydroxychloroquine) *n* = 12	6	15	Day 1 favipiravir ~ 23 mg/kg in the morning and evening; 2–5. Days: 8.5 mg/kg via gavage in the morning and evening; day 1 hydroxychloroquine 10 mg/kg morning and evening; 2–5. Days: 3 mg/kg morning and evening (15 days)
	6	30	Day 1 favipiravir ~ 23 mg/kg in the morning and evening; 2–5. Days: 8.5 mg/kg via gavage in the morning and evening; day 1 hydroxychloroquine 10 mg/kg morning and evening; 2–5. Days: 3 mg/kg morning and evening (30 days)

In the 1st group, on the 1st day, favipiravir was ~ 23 mg/kg in the morning and evening; 2–5. On days, 8.5 mg/kg was administered via gavage in the morning and evening. 2. Group 1. Day 1: Hydroxychloroquine 10 mg/kg in the morning and evening; 2–5. On days, 3 mg/kg was administered by gavage in the morning and evening. Group 3 was given favipiravir at ~ 23 mg/kg in the morning and evening on the 1st day; 2–5. On days, 8.5 mg/kg was administered in the morning and evening by gavage; on day 1, 10 mg/kg of hydroxychloroquine was administered in the morning and evening; and on days 2–5. On days, 3 mg/kg was administered by gavage in the morning and evening (Caroline et al. 2014; Hanioka et al. 2021; Masimirembwa et al. 1994).

In the radiological evaluation of our study, anteroposterior and lateral femur radiographs were taken on the 15th and 30th days after all treated femurs were sacrificed from the rats. In our study, fracture healing was evaluated histopathologically using the scoring system suggested in the literature. Each group was divided into two groups, each with six rats, to be euthanized on the 15th and 30th days (Huo et al. 1991).

### Surgical technique

**Fig. 1:**
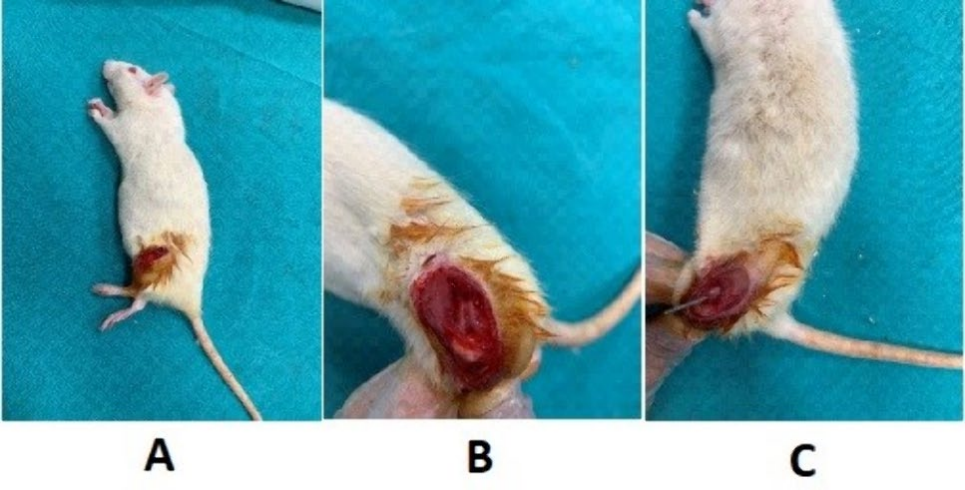
The procedure for the surgical protocol. **A** Anteromedial 1.5-cm longitudinal incision anteromedially through the skin and opening the joint capsule medial to the patella. **B** After anteromedial longitudinal incision of the knee, the patella is tilted laterally to expose the femoral intercondylar region. **C** A 1.2-mm Kirschner wire is inserted between the femoral condyles to exit the proximal region of the femur

The rats for which the necessary follow-up and preparations were made were taken to the intervention room. The anesthetic drug dose was calculated by weighing the weight of each rat with an electronic scale. The combination of 50 mg/kg ketamine and 10 mg/kg xylazine was used as an anesthetic. Anesthesia was administered intraperitoneally from the left inguinal region. After the right knees of the rats were shaved, they were stained with povidone iodine. Anteromedially, the skin was passed through a 1.5-cm longitudinal incision. The joint capsule was opened from the medial side of the patella. The patella was tilted laterally, and the knee was flexed. The intercondylar region of the lower end of the femur was exposed. A 1.2-mm Kirschner wire was placed between the femoral condyles such that it protruded from the proximal region of the femur. The wire remaining in the canal was cut at the level of the femoral condyles so that it would not protrude from the condyle (Fig. 1).

In the group whose periosteum was excised, in addition to this procedure, the femur was exposed with a lateral longitudinal incision, and its periosteum was excised with the help of a scalpel. The patella was reduced by extending the knee. The capsule was sutured with Vicryl. The skin was closed with silk. Subsequently, the wound site was wiped with povidone iodine using the method described by Einhorn (1998). Accordingly, the right femur of each animal was placed in a specially made blunt guillotine system consisting of four parts: the base part, the part where the animal was placed, the guillotine part, and the weight part. The adjusting screws of the guillotine system were adjusted to allow only 1.5 mm of movement of the blunt guillotine. A closed fracture was created by letting a 500-g weight fall freely from a height of 35 cm (Einhorn 1998; Lane & Sandhu 1987).

### Histopathological evaluation

Soft tissues covering all fractured femurs were stripped without removing the periosteum, and the K-wire was carefully removed without damaging the callus tissue. Before decalcification in 7% formic acid, the femurs were fixed in 4% paraformaldehyde at 4 °C for 48 h.

After decalcification, the samples were embedded in a paraffin block, and 7-µm sections were cut. The sections were stained with hematoxylin and eosin dye. Fracture healing was evaluated histopathologically using the scoring system proposed by Huo et al. (1991).

### Radiological evaluation

**Fig. 2:**
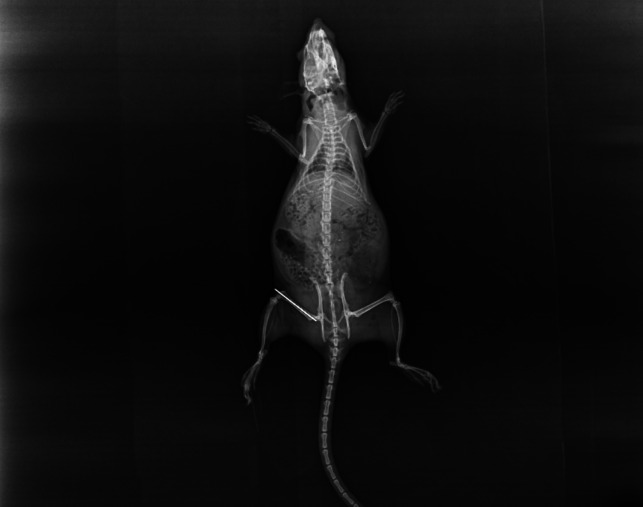
The control radiograph on postoperative day 0

Anteroposterior and lateral femur radiographs were taken on the 15th and 30th days for all treated femurs that were sacrificed from the rats (Fig. 2).

Lane and Sandhu’s grading system was used for radiological scoring (Lane & Sandhu 1987). Scoring was evaluated by two separate orthopedists independent of the study.

### Statistical analysis

In this study, statistical analyses were performed with the NCSS (Number Cruncher Statistical System) 2007 Statistical Software (UT, USA) package program. In addition to descriptive statistical methods (mean, standard deviation), the distribution of variables was examined with the Shapiro–Wilk normality test, one-way analysis of variance was used in intergroup comparisons of normally distributed variables, Tukey multiple comparison test in subgroup comparisons, independent *t*-test in comparisons of paired groups, and chi-square test in comparisons of qualitative data. The results were evaluated at a significance level of *p* < 0.05.

## Results

A statistically significant difference was observed between the mean histologic scores of favipiravir, hydroxychloroquine, favipiravir + hydroxychloroquine, and control groups on day 15 (*p* = 0.0001). The hydroxychloroquine group was found to be statistically significantly higher than the 15th day histologic scoring averages of hydroxychloroquine and favipiravir + hydroxychloroquine groups (*p* = 0.001, *p* = 0.001), whereas the control group was found to be significantly higher than the 15th day histologic scoring averages of favipiravir and favipiravir + hydroxychloroquine groups. Day 15 histologic scoring averages of favipiravir and favipiravir + hydroxychloroquine groups (*p* = 0.043, *p* = 0.023), while no statistically significant difference was observed between the 15th day histologic scoring averages of the other groups (*p* > 0.05). No statistically significant difference was observed between the 30th day histologic scoring averages of favipiravir, hydroxychloroquine, favipiravir + hydroxychloroquine, and control groups (*p* = 0.887). The 30th day histologic scoring averages of the favipiravir group were statistically significantly higher than the 15th day histologic scoring averages (*p* = 0.0001). No statistically significant difference was observed between day 15 and day 30 histologic scoring averages of the hydroxychloroquine group (*p* = 0.787). The 30th day histologic scoring averages of the favipiravir + hydroxychloroquine group were statistically significantly higher than the 15th day histologic scoring averages (*p* = 0.001). No statistically significant difference was observed between day 15 and day 30 histologic scoring averages of the control group (*p* = 0.088) (Tables 2 and 3).

**Table 2:** One-way variance analysis values and independent *t*-test of histological values of favipiravir, hydroxychloroquine, favipiravir + hydroxychloroquine, and control groups on days 15 and 30

		Favipiravir group *n* = 6	Hydroxychloroquine group *n* = 6	Favipiravir + hydroxychloroquine group *n* = 6	Control group *n* = 6	*p*‡
Histological scoring	15 days	4.33 ± 0.52	7.00 ± 0.89	4.17 ± 0.75	6.00 ± 1.52	**0.001**
	30 days	7.83 ± 1.47	7.33 ± 2.81	8.17 ± 1.17	7.67 ± 1.51	0.887
	p*	**0.0001**	0.787	**0.0001**	0.088	

^‡^One-way analysis of variance

^*^Independent *t*-test, *p* < 0.05

**Table 3:** Tukey multiple comparison values of histological values of favipiravir, hydroxychloroquine, favipiravir + hydroxychloroquine, and control groups on days 15 and 30

Tukey multiple comparison test	*p*
Favipiravir group/hydroxychloroquine group	**0.001**
Favipiravir group/favipiravir + hydroxychloroquine group	0.991
Favipiravir group/control group	**0.043**
Hydroxychloroquine group/favipiravir + hydroxychloroquine group	**0.001**
Hydroxychloroquine group/control group	0.338
Favipiravir + hydroxychloroquine group/control group	**0.023**

*p* < 0.05

**Fig. 3:**
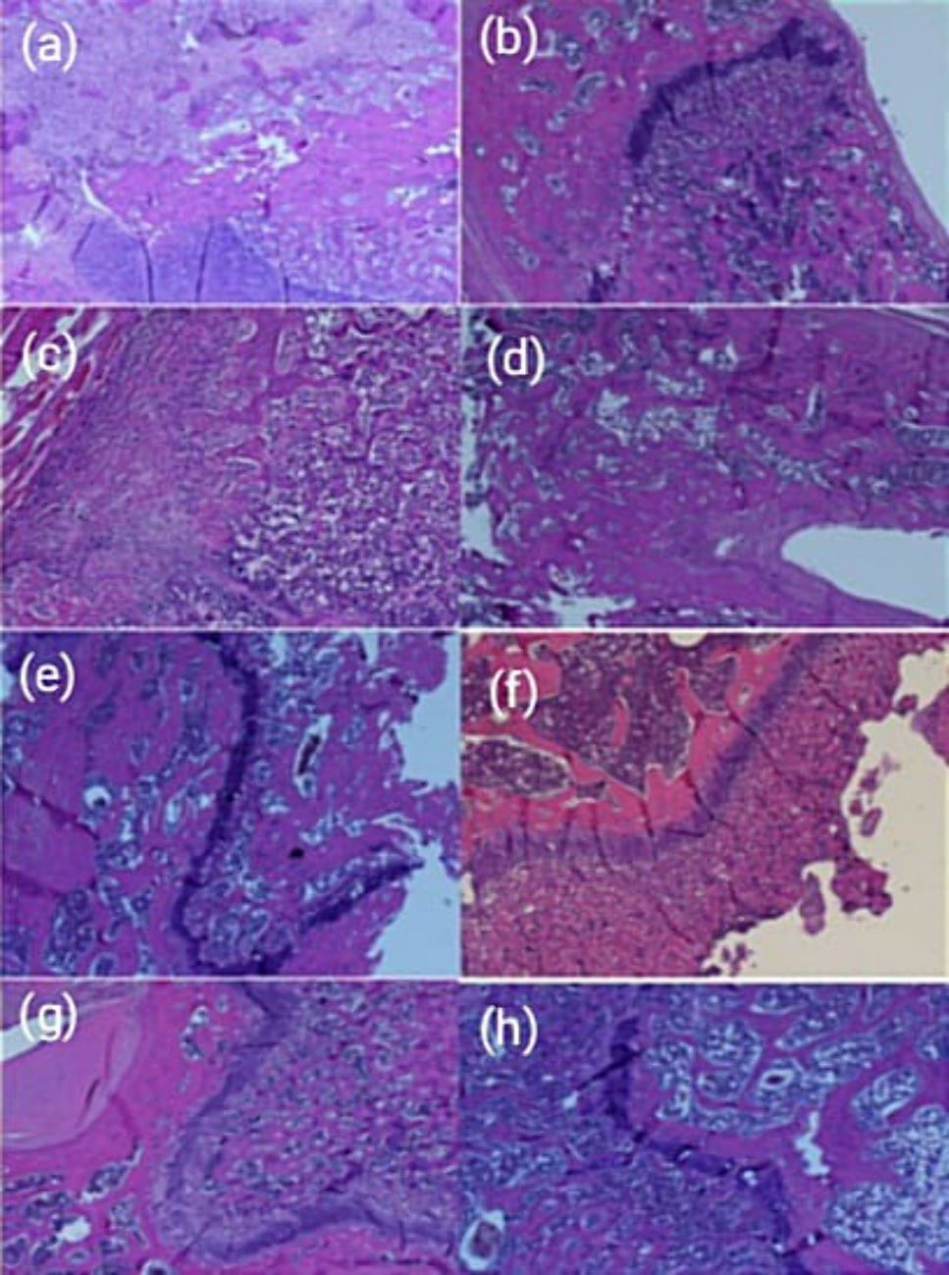
Microscopy images of the histopathological appearance of the callus in the 15-day and 30-day groups. **a** A microscopic image of the histopathologic appearance of the callus at day 15 in the favipiravir group. Fibrous tissue and cartilaginous tissue are seen (score 3) (HE, × 100). **b** A microscopic image from the histopathologic appearance of the callus at day 30 in the favipiravir group. Densely immature bone is seen (score 8) (HE, × 4). **c** A microscopic image from the histopathologic appearance of the callus at day 15 in the hydroxychloroquine group. Completely immature bone is seen (score 8) (HE, × 4). **d** A microscopic image from the histopathologic appearance of the callus at day 30 in the hydroxychloroquine group. Mature (lamellar) bone is seen (score 10) (HE, × 4). **e** A microscopic image from the histopathologic appearance of the callus at day 15 in the control group. It shows predominantly cartilage and a small amount of immature (woven) bone (score 5) (HE, × 4). **f** A microscopic image of the histopathologic appearance of the callus at day 30 in the control group. Equal proportions of cartilage and immature bone are seen (score 6) (HE, × 4). **g** Microscopic image from the histopathologic appearance of the callus at day 15 in the favipiravir + hydroxychloroquine group. Equal proportions of cartilage and immature bone are seen (score 6) (HE, × 4). **h** A microscopic image from the histopathologic appearance of the callus at day 30 in the favipiravir + hydroxychloroquine group. Equal proportions of cartilage and immature bone are seen (score 6) (HE, × 4)

Microscopy images of the histopathological appearance of the callus in the 15-day and 30-day groups are also shown in Fig. 3.

No statistically significant difference was observed between day 15 radiologic scoring distributions of favipiravir, hydroxychloroquine, favipiravir + hydroxychloroquine, and control groups (*p* = 0.330). No statistically significant difference was observed between day 30 radiologic scoring distributions of favipiravir, hydroxychloroquine, favipiravir + hydroxychloroquine, and control groups (*p* = 0.618). The 30th day radiologic scoring distributions of the favipiravir group were found to be statistically significantly higher than the 15th day radiologic scoring distributions (*p* = 0.007). No statistically significant difference was observed between day 15 and day 30 radiologic scoring averages of the hydroxychloroquine group (*p* = 0.055). In the favipiravir + hydroxychloroquine group, the 30th day radiologic scoring fracture line disappearance and complete bone fusion distributions were statistically significantly higher than the 15th day radiologic scoring distributions (*p* = 0.007). No statistically significant difference was observed between day 15 and day 30 radiologic scoring averages of the control group (*p* = 0.273) (Table 4).

**Table 4:** Chi-square test values of radiological values of favipiravir, hydroxychloroquine, favipiravir + hydroxychloroquine, and control groups on days 15 and 30

Radiological scoring		Favipiravir group		Hydroxychloroquine group		Favipiravir + hydroxychloroquine group		Control group		*p* +
15 days	**Callus formation**	5	83.33%	2	33.33%	3	50.00%	4	66.67%	0.330
	**Onset of bone boil**	1	16.67%	4	66.67%	3	50.00%	2	33.33%	
30 days	**No recovery**	0	0.00%	1	16.67%	0	0.00%	0	0.00%	0.618
	**Callus formation**	0	0.00%	1	16.67%	0	0.00%	0	0.00%	
	**The broken line starts to disappear**	4	66.67%	2	33.33%	4	66.67%	3	50.00%	
	**Complete bony union**	2	33.33%	2	33.33%	2	33.33%	3	50.00%	
	***p***^**+**^	**0.007**		0.055		**0.007**		0.273		

^+^Square chi-square test, *p* < 0.05

**Fig. 4:**
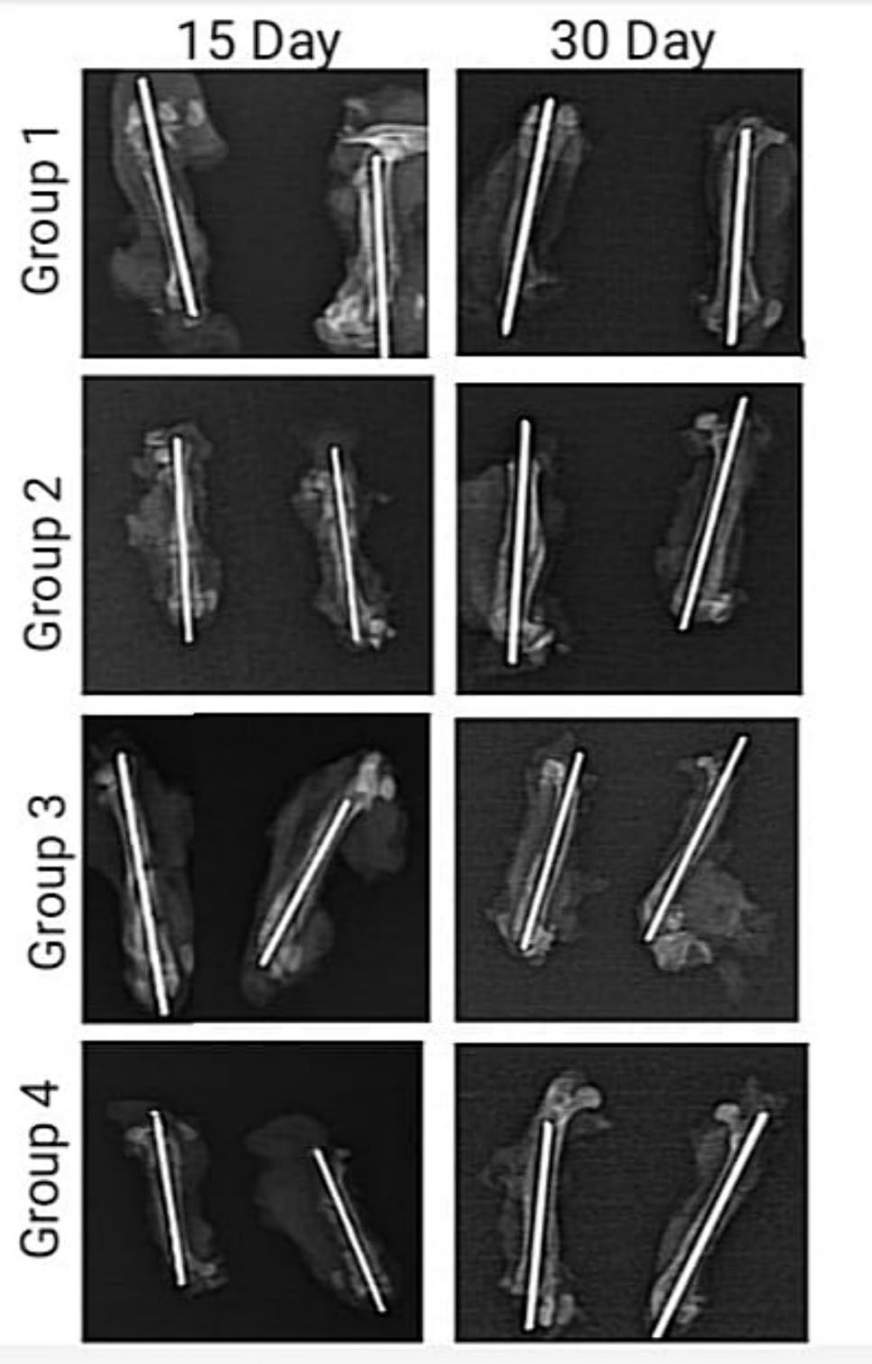
Control radiographs after surgery

The control radiographs of the groups at 15 days and 30 days after surgery are shown in Fig. 4.

## Discussion

In this study, we investigated the effects of favipiravir and hydroxychloroquine, which can be used to treat COVID-19 and viral diseases, on fracture union in male rats radiologically and histopathologically. The results showed that favipiravir promoted fracture healing. However, no significant improvement was observed in the hydroxychloroquine group.

The ability of favipiravir to promote fracture healing may be due to its antiviral properties. Favipiravir is known to be an effective antiviral drug against RNA viruses, and this study suggested that this drug may also favorably affect the bone healing process. However, caution should be exercised about the direct applicability of the findings obtained in rats in this study to humans. It should be noted that there are differences between the biological structures of rats and humans. Therefore, further clinical studies are needed to determine how favipiravir affects fracture healing in humans.

Fracture healing is one of the most frequently discussed topics in orthopedics and traumatology. This topic is still an area of intense research. It should not be overlooked that the biology of bone fracture healing is a rapidly developing field (Byun et al. 2023; Saul & Khosla 2022; Saul et al. 2023). Advances in experiments with mouse rats have made tissue- and cell-specific skeletal regeneration research possible. As an example, it was only recently understood that chondrocytes transform into osteoblasts during bone healing and only a few years ago seminal publications conclusively reported that the periosteum and endosteum are the most important tissues that contribute to bone-forming cells during regeneration (Bahney et al. 2019). The effects of drugs that are frequently used in the field of orthopedics and traumatology on fracture healing are important in clinical and animal experiments (Fischer et al. 2018; Kurmis et al. 2012; Xiao et al. 2024).

The global pandemic of novel coronavirus disease 2019 (COVID-19) caused by severe acute respiratory syndrome coronavirus 2 (SARS-CoV-2) began in December 2019 and has since spread worldwide. In another study conducted during the pandemic, Egol et al. retrospectively and prospectively evaluated the mortality and complications of hip fracture in 115 patients among 138 patients during the COVID-19 pandemic. They found that those with confirmed or suspected COVID-19 infection had a significantly increased risk of death after hip fracture (Egol et al. 2020).

Favipiravir is a drug that could be used in the fight against COVID-19. This drug is a pyrazine carboxyamide derivative with antiviral activity against various RNA viruses (such as influenza virus, rhinovirus, and respiratory syncytial virus) (Furuta et al. 2013).

According to the results of our study, the positive effect of favipiravir on fracture healing can be explained by various mechanisms. Favipiravir is an antiviral drug effective against various RNA viruses, especially influenza virus. This antiviral activity affects the course of the disease by suppressing or stopping virus replication. Infections caused by viruses can affect the response of the immune system and thus a number of events at the cellular and molecular levels (Rocha-Pereira et al. 2012). The antiviral effect of favipiravir may favorably influence this response by modulating the inflammatory response associated with infection and inhibiting virus replication. Favipiravir may have a potential immunomodulatory effect on the immune system (Oestereich et al. 2014). Immunomodulators may reduce or increase inflammation by regulating the immune system. Fracture healing is a complex process in which inflammatory and immune cells are involved at various stages. Therefore, the effects of favipiravir on the immune system may affect fracture healing (Indari et al. 2021). The anti-inflammatory effect of favipiravir may also promote fracture healing. Inflammation is an important part of the healing process, but excessive or chronic inflammation can negatively affect fracture healing. The potential of favipiravir to control inflammation may accelerate fracture healing. The positive effects of favipiravir on fracture healing may be related to the modulation of a number of events at the cellular and molecular levels. This drug may affect the cell cycle, protein synthesis, and other biological processes. These effects may have a potential impact on the proliferation, differentiation, and maturation of bone cells via certain cellular functions and molecular pathways, both of which play a role in immune activation, in part by accumulating in the endosomes/phagosomes of cells (Yao et al. 2020).

Hydroxychloroquine, another agent we used in our study, is an aminoquinoline that has been used to treat malaria for 50 years. It has been used for many years in the treatment of autoimmune diseases such as rheumatoid arthritis due to its immunomodulatory effects as well as its antimalarial effects. According to the results of our study, no significant healing was observed in the hydroxychloroquine group. These results suggest that hydroxychloroquine may have no effect on fracture healing in rats. However, further research is needed to assess how these results translate to humans. The lack of the expected effect of hydroxychloroquine on fracture healing can be explained by several reasons. Like favipiravir, hydroxychloroquine may not have direct bone healing-promoting effects. This drug may not have been designed to directly accelerate bone healing, especially when used to treat autoimmune diseases and certain infections. The way hydroxychloroquine is metabolized in the body and its mechanism of action may be different from those of other drugs, such as favipiravir. Therefore, hydroxychloroquine may not have had the expected effect on fracture healing. The dose and method of administration may also have affected the effect. The dose or method of administration of hydroxychloroquine used in our study may not have been sufficient to show an optimal effect. Dosages can significantly affect the efficacy of drugs, and it is therefore important to determine appropriate doses.

The number of studies investigating the effects of hydroxychloroquine on fracture healing is limited in the literature, and there are generally few studies focusing on this topic (Önaloğlu et al. 2024; Topak et al. 2023). Therefore, it may be difficult to reach a clear conclusion regarding the effects of hydroxychloroquine on fracture healing in the current literature. A recent study in the literature on rats reported that oral hydroxychloroquine intake impairs the fracture-healing process by causing oxidative stress in rats (Topak et al. 2023). However, more biomolecular research is needed to understand the mechanism underlying these effects.

### Study limitations

The limitations of the study are as follows: Biomechanical and biochemical evaluations could not be made. Another limitation is the short follow-up period. Additionally, the limited number of rats used in the study may limit the generalizability of the results. Therefore, larger-scale human studies are needed in the future.

## Conclusion

The results of our study show that favipiravir has a supportive effect on fracture healing and hydroxychloroquine is ineffective in increasing this positive effect. However, more research is needed.

## Data Availability

The datasets used and/or analyzed during the current study are available from the corresponding author on reasonable request.
